# Daughters-in-law and mothers-in-law in lowland rural Nepal: The balance of power and health outcomes

**DOI:** 10.1093/emph/eoag006

**Published:** 2026-03-27

**Authors:** Akanksha A Marphatia, Laura Busert-Sebela, Dharma S Manandhar, Alice Reid, Mario Cortina-Borja, Naomi Saville, Meenakshi Dahal, Mahesh C Puri, Jonathan C K Wells

**Affiliations:** Population, Policy and Practice Research and Teaching Department, Great Ormond Street Institute of Child Health, University College London, London, UK; Department of Disease Control, Environmental Health Group, London School of Hygiene and Tropical Medicine, London, UK; Population, Policy and Practice Research and Teaching Department, Great Ormond Street Institute of Child Health, University College London, London, UK; Chair of Public Health Nutrition, University of Bayreuth, Bayreuth, Germany; Mother and Infant Research Activities, Kathmandu, Nepal; Department of Geography, University of Cambridge, Cambridge, UK; Population, Policy and Practice Research and Teaching Department, Great Ormond Street Institute of Child Health, University College London, London, UK; Institute for Global Health, University College London, London, UK; Center for Research on Environment Health and Population Activities, Kathmandu, Nepal; Center for Research on Environment Health and Population Activities, Kathmandu, Nepal; Population, Policy and Practice Research and Teaching Department, Great Ormond Street Institute of Child Health, University College London, London, UK

**Keywords:** daughter-in-law, mother-in-law, power, mental health, conflict, nutritional status

## Abstract

**Background and objectives:**

The relationship between daughters-in-law (DIL) and mothers-in-law (MIL) is often portrayed as conflictual, though from an evolutionary perspective, the two women could cooperate to gain Darwinian fitness benefits. We investigated this relationship in a society where it is central to women’s social niche. Without asking directly, we aimed to evaluate markers of conflict and harmony in households, and whether variability in this balance was reflected in differential health outcomes. We also tested whether conflict was less likely in households with more children, and hence greater shared genetic interest.

**Methodology:**

We studied 110 co-resident DIL–MIL dyads from rural lowland Nepal and compared anthropometry, physical and mental health, lived experience, agency, and attributed contributions to household tasks.

**Results:**

Overall, DIL had better mental health than MIL, and greater weight and height. Tension over childcare and resting was common. Mental health and height differences within dyads were greater in households with inferred conflict over childcare, compared to inferred harmony households. DIL had better health outcomes and higher weight and height relative to MIL if they lived in conflict compared to harmony households, whereas MIL had better health outcomes if they lived in harmony households. Number of children did not predict conflict.

**Conclusions and implications:**

Despite MIL being senior in the household, DIL reported lower stress and better mental health, especially in households with inferred conflict. DIL in such households also had better nutritional status. Having had time since marriage to consolidate their status in the household, DIL may have increased their agency and power.

**Lay summary:**

Our study in rural Nepal aimed to identify which of daughter-in-law or mother-in-law held the upper hand in the household hierarchy, and how this was reflected in health outcomes. Although mothers-in-law are more senior, daughters-in-law had better physical and mental health, especially in households with inferred conflict over childcare.

## INTRODUCTION

The balance of cooperation and conflict within families or households has long attracted attention from evolutionary biologists. According to Hamilton’s law, we may expect greater levels of cooperation among individuals with higher levels of genetic relatedness [1]. However, cooperation may also occur among household members who are genetically unrelated, most obviously two parents, who share Darwinian fitness interests in raising their offspring [2]. In extended families, households may include either the paternal or maternal grandparents, further bringing relationships between in-laws into the picture. In lowland Nepal, for example, where our research is conducted, a wife typically moves upon marriage into her husband’s household and co-resides with his parents.

Following Hamilton’s law, we might expect low levels of cooperation among unrelated in-laws, and that in patrilocal societies, senior household members might aim to control and exploit the daughter-in-law to maximize the reproductive fitness of their son. This prediction has some support in the literature [3–9]. However, a counter-balancing perspective is that affinal relatives may also cooperate [10–12], through their shared interests in the fitness of any offspring born into the household [13]. Since women often have the primary responsibility for childcare, examining the relationship between co-resident daughters-in-law (DIL) and mothers-in-law (MIL) may represent a particularly informative approach to evaluating household cooperation versus conflict through an evolutionary lens. Both DIL and MIL share genetic interest in the children of the DIL, with average relatedness, respectively, of 50% and 25%.

A recent analysis of different household residence patterns in Chinese societies by Chen et al. found that women performed more household work than men, and that this was particularly the case when women dispersed from the natal home after marriage [14], as is the case in our study population. Our own focus is on unrelated women with different ages and statuses within the same household. In patriarchal patrilocal societies, the implications of women migrating into the household change profoundly by age: every incoming woman is initially a DIL with low status after marriage, but if she produces a son she becomes a MIL with higher status later in life. The nature of the MIL–DIL relationship is thus inherently one of asymmetry in agency and roles; moreover, the consequences of this asymmetry may also vary across households.

Whether MIL and DIL cooperate or show markers of conflict may depend on the number of children in the household and their sex. The greater the number of children, the more DIL and MIL share common interests in the Darwinian fitness of their offspring and grandoffspring, respectively. Moreover, the sex of the children may also be relevant. Paternal grandmothers are more closely genetically related to their granddaughters than to their grandsons, as X-linked genes of the MIL have a 25% chance of passing to the granddaughter, but 0% chance of passing to the grandson [15]. However, because daughters in this society are married out of the household during adolescence whereas sons tend to remain, grandsons may potentially provide better fitness pay-offs for MILs, as they are likely to care for her sons as they age and become grandfathers.

A genetic conflict of interest between household members may not necessarily translate into physical conflict, but is predicted to generate ‘conflicts of interest’ that permeate many aspects of daily life. Markers of conflicts of interest may be identified in behaviour, physiology, or health outcomes [16, 17]. Recently, Daly and Perry described an elevated risk of mortality due to conflict between DIL and MIL in Bangladesh as a two-way street, adversely impacting both partners [4].

Building on their approach, we aimed to characterize the nature of the DIL–MIL relationship and its association with health outcomes in a Maithili-speaking population in lowland Nepal. In this society, DIL tend to have little opportunity to spend time or make social connections outside the household, meaning that relationships among household members dominate their social niche. Younger DIL generally have the least decision-making power in the household, and perform more unpaid care activity, overseen by the MIL [18, 19]. The quality of the DIL–MIL relationship has acquired new importance in this setting, because the DIL’s husband has often migrated out of the country for work [19, 20]. The MIL–DIL relationship may also evolve over time, as both women gain age and experience and the DIL gains confidence to assert herself in the household. In other contexts, taller women have been proposed to be less submissive and hence to have greater agency in household dynamics [21]. It is possible therefore that taller DIL could be more assertive than their shorter peers.

Physical health is generally considered to decline with age, as physiological ‘wear-and-tear’ accumulates. In high-income settings, older people often exhibit muscle loss, cardiometabolic decline and poorer appetite compared to younger people, though these trends may vary in other ecological settings [22–25]. On the other hand, increasing seniority might give older individuals priority access to resources that are beneficial for health, including food and the labour of other members of the household, and their physical workload may potentially decline. The relationship between mental health and age is also variable across populations. The notion of a universal dip in psychological human well-being in midlife is not supported by evidence from non-industrialized societies. In Asian populations, subjective well-being increases until old age, and in Nepal, there was a linear increase in life satisfaction with age across the adult lifespan [26]. This pattern might relate in part to the household hierarchies outlined above; however, this association has received little attention. How the change in status from DIL to MIL shapes the effects of age on health, and the introduction of a new DIL into the household, therefore merits investigation.

In a previous analysis of data collected on the same cohort as investigated here, we evaluated the mental health and autonomy of DIL in 444 households, according to the presence or absence of both her husband and her parents-in-law [19]. We found that DIL co-residing with parents-in-law but not husband had better mental health, indicative of greater social support, but also reduced autonomy, indexed by diminished household decision-making and bargaining power. Conversely, DIL in nuclear households and co-residing with their husband but not parents-in-law had greater autonomy, but poorer mental health. This study demonstrated a trade-off between relationships of control and support for DIL, but did not provide information on the health of other women in the household.

### Objectives

**Figure 1:**
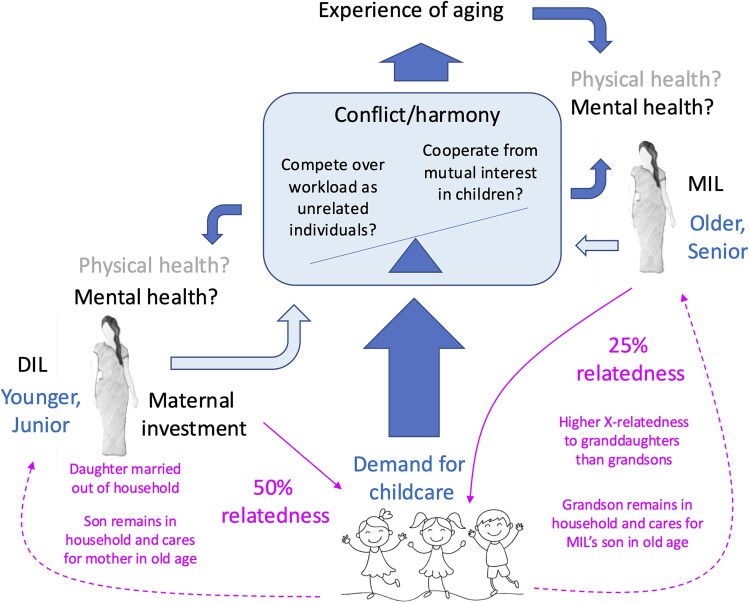
Conceptual diagram illustrating how the relationship between the daughter in law (DIL) and mother-in-law (MIL) may shape their physical and mental health. From an evolutionary perspective, DIL and MIL may cooperate through their shared interest in the Darwinian fitness of the DIL’s children (thin arrows); however, they may also compete over the burden of work in the household, generating tension. The outcome of this cooperation-conflict balance may then relate to the mental and physical health of the two women, and potentially impact the experience of ageing in the DIL. Whether or not the women compete or cooperate may further be influenced by the number of children in the household, which determines both the magnitude of shared fitness interest, but also the demand for childcare. The dotted arrows indicate how the sex of children has fitness implications for both DIL (mother) and MIL. MIL share X-related genes with granddaughters but not with grandsons. However, granddaughters will eventually be married into other households, whereas grandsons will remain as adults, and will likely care for both their mother (current DIL) and the MIL’s son (DIL’s husband) as they age and invest in their own grandchildren. Child image designed by Freepik.

To break new ground, we now investigate household activities and health outcomes in co-resident DIL–MIL dyads in 110 households in the same population. Importantly, every DIL in this society is likely to become a MIL at a later age if she produces a son, though declining fertility and an increase in nuclear households constrain this likelihood. As in other traditional societies, women perform a variety of activities that are vital for both household subsistence and Darwinian fitness (e.g. farmwork, childcare), but which are typically financially unremunerated and assigned low status. This may lead to tension over the contributions of DIL and MIL to the household livelihood and the burden of unpaid work. Without directly asking DIL and MIL about any conflict between them, we aimed to evaluate their experience of stress, their opportunity to gain adequate nutrition at household meals, their nutritional status, and their perspective of each other’s roles in household subsistence and unpaid care activities. Using this approach, we aimed to identify which of DIL or MIL was more vulnerable to poor health in this setting, which, in broad terms, could indicate the ‘balance of power’ between them (Fig. 1). We also examined whether this tension was associated with the number of children in the household, which shapes the demand for childcare.

We tested the following hypotheses:

1. MIL (more senior) experience overall better nutritional status but poorer physical health than DIL.
2. MIL (more senior) experience overall better mental health than DIL.
3. DIL and MIL may disagree over who performs more of specific household tasks.
4. The greater the level of disagreement, the greater the stress among both women.
5. The level of disagreement is associated with disparity in body size and nutritional status.
6. Households with more DIL children, and in particular more daughters, have lower risk of conflict.

## METHODS

### Study setting

The longitudinal Growth Monitoring Study (GMS) in Dhanusha district in Madesh Pradesh (Terai region) in Nepal began as a birth cohort in 2012 with the recruitment of 602 infants, who were then followed every 28 days until 2 years of age, at ~6 years (2018) [27], and in a subsample of 200 households at ~8 years (2021). We focus on the 110 co-resident DIL–MIL pairs from 2021. These women are likely to be representative of the whole cohort, as our previous analysis found minimal differences in the traits of co-residing DIL/MIL pairs vs DIL living in nuclear households (which often emerged after the MIL died) [19].

Data collection in 2021 was conducted by four female researchers from the Centre for Research on Environment, Health and Population Activities (CREHPA) in Nepal. Researchers were trained to administer oral questionnaires to co-resident DIL–MIL pairs. To ensure confidentiality, questionnaires were administered to the two women simultaneously, but in separate locations in their homes.

The Nepal Health Research Council for (95/2013, 13/2018) and University College London (11 345/001) granted research ethics for the original GMS. As this cohort study began before federalization in 2012, Village Development Committee Secretaries consented to the inclusion of geographic clusters. Research ethics approvals for the 2021 study were granted by the Nepal Health Research Council (28/2020P), University College London (0326/016), and University of Cambridge (1403). Participants provided written consent.

### Data

Household-level data on caste-ethnicity, wealth, and food security were collected at the 2018 follow-up. Wealth reflected context-specific household infrastructure and ownership of material goods [28], as routinely collected by Demographic Health Surveys (Supplementary Text S1) [29]. Food security was assessed using the Household Food Insecurity Access Scale, which included nine questions, each with scores of 0–3 (Supplementary Text S1) [30].

Demographic characteristics collected in 2021 included age (y), current marital status (married, separated, or widowed), marriage age (y), number, and sex of the DIL’s living children, years of school completed (y) in integer years, height (Shorr board, metres (m)), weight (Seca 803 electronic scales, kilograms (kg)), and body mass index (BMI, kg/m^2^).

Dyads self-rated their overall physical health (very poor, poor, good, and very good), whether they had a poor appetite (yes vs no), had low energy/slowed down (yes vs no), and had been ill to the extent of losing weight (yes vs no). As it is customary for the cook (often the junior DIL in the household) to eat last in most parts of Nepal, women were asked which family members ate dinner after them [31].

Eleven questions assessed the lived experience of DIL and MIL in the 4 weeks before the survey (Supplementary Text S2). To provide a holistic assessment of mental well-being, a set of questions was collated from questionnaires previously administered in Nepal in our study population. This approach was chosen because we were not trying to diagnose a clinical pathology such as depression, but rather assess overall well-being across different aspects of women’s lives. Given low levels of literacy, binary responses (yes/no) were used, as previously in our study population [32, 33]. We summed these 11 questions on lived experience to create an overall stress score, ranging from 0 to 11, with 0 indicating no stress and 11 extreme stress.

Effort on subsistence work was assessed by asking both women about the time spent performing five types of unpaid care tasks: tending to family’s crops and animals, fetching water, housework, childcare, and resting. Women could give one of four answers: the other spent more time; both spent equal time; they spent more time than the other on the task; or the task was not performed in their household.

### Data coding

Following other studies conducted on this population [34], caste/ethnicity was coded into three groups: disadvantaged (Dalit, Muslim), mid (Janajati, other Terai), or advantaged (Sudi, Yadav, and Brahmin). A wealth index was constructed using principal component analysis based on household infrastructure and material assets (Supplementary Text S1), and coded into quartiles (poorest = 1, richest = 4). The food security measure (secure, mildly, moderately, or severely insecure; Supplementary Text S1) was aggregated to a binary outcome, food secure vs any food insecurity, based on the distribution of these data in our sample.

The overall stress score was expressed in whole integers ranging from 0 to 11. For statistical analysis, we also conducted a principal component analysis of the 11 lived experience questions (Supplementary Text S2), which resulted in a continuous score that predominantly reflected lost sleep (factor loading 0.947) and anxiety (factor loading 0.940), which we interpreted as stress.

Education was categorized into four levels reflecting the education system in Nepal: none, primary (grades 1–5), lower-secondary (grades 6–8), or secondary/higher (grades 9 onwards). Regression models controlled for a binary education variable (none vs any) because most MIL had no education. Women’s overall self-rated physical health was coded as poor, good, or very good, and then recoded as a binary variable (poor, vs good or very good) for statistical analysis based on the distribution of these data in our sample.

To evaluate the sex ratio of the DIL’s total live children, we added 1 to each of the number of sons and number of daughters, so that no child category was zero. We then divided the adjusted daughter number by the adjusted son number to calculate the female–male sex ratio.

Responses to effort-related questions were cross-tabulated across DIL and MIL responses to indicate *perceptions* of who spent time on each of the five tasks. Aggregate household scores were computed for dyads on overall inferred harmony (the dyad expressed either agreement or generosity when attributing effort), or inferred conflict (the dyad disagreed over perceived effort), for each of the five tasks. We then examined, for the tasks where inferred conflict was common, whether its occurrence was associated with particular characteristics of DIL or MIL (age, age at marriage, and education), household wealth or food security. In the rest of the article, conflict and harmony refer to *inferred* conflict and harmony.

Finally, we examined whether conflict was associated with variability in the stress score, anthropometry, or self-rated physical health for each woman. We first compared DIL against MIL, separately for conflict and harmony households, to see which member of the dyad had better outcomes in each type of household. We then compared DIL in conflict households versus their counterparts in harmony households (and likewise for MIL), to analyse what factors were associated with conflict within each type of relative.

### Statistical analysis

Household and demographic characteristics were summarized using mean and standard deviation (SD) and frequencies (number, %). DIL–MIL differences were tested using paired samples *t*-tests (95% confidence interval, CI) and chi-squared tests. Lived experience of the groups was compared through odds ratios (OR, 95% CI) from chi-squared tests, using MIL as the reference category. A similar approach was used to compare lived experience of DIL and MIL in conflict vs harmony households (i.e. mismatched vs matched) perceptions of who does what work on a given task, using harmony households as the reference.

To address possible confounders, we constructed multivariable linear regression models for continuous outcomes and multivariable logistic regression models for binary outcomes. We adjusted for household wealth (coded as quartiles) and education level (coded as a binary variable, none vs any) at the individual level. It was not possible to investigate age at the individual level because age and MIL/DIL status have a correlation of 0.92, and hence would cause multicollinearity and could not usefully be dissociated. Likewise, because of secular trends in marriage age, this variable was strongly correlated with MIL/DIL status. Therefore, for age of MIL, age of DIL, marriage age of MIL, and marriage age of DIL, we generated four variables that were assigned at the dyad level, i.e. each member of a dyad was assigned the same value (e.g. for DIL age). In this way, we could adjust separately for variability in DIL or MIL age or marriage age.

To assess whether the number or sex of the DIL’s living children predicted conflict, we fitted logistic regression models. Separate models were used to evaluate as predictors the total number of children, the numbers of sons and daughters, and the sex ratio of the composite child sample. These models adjusted for the same covariates listed above.

In all models, we focused on understanding the magnitude of the effects but report *P*-values for information.

Colour-coded heatmaps were used to visualize agreement (harmony or generosity) versus potential conflict in agency between the two women, inferred by their responses regarding the time spent on each task.

Statistical analyses were conducted in SPSS 27 (IBM Corp., Armonk, NY).

## RESULTS

About a third of households were from disadvantaged, mid, and advantaged caste groups, respectively. Nineteen percent were poor households, with the rest equally spread over other wealth categories. A quarter of households faced some degree of food insecurity. Dyads had co-resided on average for 14.7 (SD 5.2) years, broadly since the DIL’s marriage 15.5 (SD 5.8) years ago.

Characteristics of the sample are given in Table 1. DIL married on average at 15 years (SD 1.7) and MIL at 12 years (SD 2.6). DIL were on average taller and heavier than MIL, but did not differ in their BMI. Most DIL were currently married, compared to 78% of MIL. The remaining MIL were either separated (2%) or widowed (20%). DILs were more educated than their MILs, most of whom had no formal education. The mean (SD) number of children of the DILs was 3.5 (0.9), with a median of 1.9 (1.0) daughters and 1.6 (0.7) sons.

**Table 1:** Individual characteristics of co-resident DIL and MIL dyads.

	DIL		MIL		Difference, DIL relative to MIL	
	Mean	SD	Mean	SD	△ (95% CI)	*P*-value^a^
Age (y)	31.2	4.2	60.2	7.6	−29.0 (−30.2, −27.8)	<.001
Age at marriage (y)	15.2	1.7	12.4	2.6	2.9 (2.3, 3.4)	<.001
Height (cm)^*^	151.5	0.49	148.2	0.51	3.3 (1.9, 4.7)	<.001
Weight (kg)^*^	49.3	8.2	45.9	10.1	3.5 (1.1, 5.8)	.005
BMI (kg/m^2^)^*^	21.5	3.3	20.8	4.1	0.7 (−0.3, 1.6)	.156

	***F***	**%**	***F***	**%**	***P*-value** ^**b**^	
Marital status					<.001	
Married	109	99	86	78		
Separated	0	0	2	2		
Widowed	1	1	22	20		
					
Formal education (y)					<.001	
None	67	61	103	94		
Primary (1–5 y)	20	18	7	6		
Lower-secondary (6–8 y)	11	10	0	0		
Secondary (≥9 y)	12	11	0	0		

*n* = 110. ^*^n = 108. y, years; DIL, daughter-in-law; MIL, mother-in-law; SD, standard deviation; △, difference, DIL relative to MIL; CI, confidence interval; cm, centimetre; kg, kilogram; m, metre; *F*, frequency; %, percentage.

a Paired *t*-test.

b Chi-squared test.

Multivariable logistic regression models showed that compared to MILs, DILs showed lower odds of reporting poor physical health, poor appetite, and weight loss, but did not differ in having low energy (Table 2). In the large majority of households, the DIL ate after the MIL.

**Table 2:** Health and eating behaviours of co-resident DIL and MIL dyads.

	DIL		MIL		Odds of DIL having outcome relative to MIL, by logistic regression			
	*F*	%	*F*	%	Model 1 Unadjusted OR (95% CI)	*P*-value	Model 2 Adjusted OR (95% CI)^a^	*P*-value
Poor physical health	11	10	20	18	0.5 (0.2, 1.1)	.085	0.5 (0.2, 1.2)	.123
								
Poor appetite	25	23	37	34	0.6 (0.3, 1.1)	.074	0.6 (0.3, 1.0)	.114
								
Low energy, slowing down	37	34	39	36	0.9 (0.5, 1.6)	.777	0.8 (0.4, 1.5)	.487
								
Ill and losing weight	9	8	27	25	0.3 (0.1, 0.6)	.002	0.3 (0.1, 0.8)	.014
								
	**DIL**		**MIL**		**Chi-squared tests for difference in frequency between DIL and MIL**			
Who eats dinner after you?	***F***	**%**	***F***	**%**	***P*-value**			
Nobody, I eat last	88	80	9	8	.001			
Husband	1	1	1	1	1.0			
– Father-in-law	0	0	0	0	NA			
– Mother-in-law	1	1	0	0	.316			
– Brother-in-law(s)	0	0	0	0	NA			
– Sister-in-law(s)	6	6	0	0	.013			
– Your daughter	2	2	1	1	.561			
– Your son	0	0	4	4	.044			
– Other children	0	0	0	0	NA			
– Daughter-in-law	0	0	92	84	.001			

*n* = 110. DIL, daughter-in-law; MIL, mother-in-law; F, frequency; %, percentage; OR, odds ratio; CI, confidence interval.

a Model 2, logistic regression models adjusting for dyad’s age and marriage age, individual woman’s education and household assets. NA, not applicable.

Table 3 describes the lived experience of DIL and MIL. Multivariable linear regression models showed that compared to MIL, DIL had a lower stress score. Compared to MIL, DIL had lower odds of losing sleep over worry, feeling nervous, being unable to cope with life and not feeling positive about the future. Similarly, compared to MIL, DIL had lower odds of being unable to ask for support at home, to feel that if they helped someone, they would not be helped back, and to have no contact with friends or family outside her marital home. However, compared to MIL, DIL were more likely to be easily discouraged by failure.

**Table 3:** Lived experience in last 4 weeks of co-resident DIL and MIL dyads.

	DIL		MIL		Difference, DIL relative to MIL, by linear regression			
	Mean	SD	Mean	SD	Unadjusted △ (95% CI)^a^	*P*-value	Adjusted△ (95% CI)^a^	*P*-value
Overall stress score (higher value = more stressed)	3.3	1.8	4.0	1.9	−0.7 (−1.2, −0.2)	.005	−0.5 (−1.0, 0.0)	.059
PCA score of stress (loses sleep, anxious)	−0.2	1.0	0.2	1.0	−0.4 (−0.7, −0.1)	.004	−0.3 (−0.6, −0.1)	.018
		
	**DIL**		**MIL**		**Odds of outcome for DIL relative to MIL, by logistic regression**			
Lived experience (response is yes)	***F***	**%**	***F***	**%**	**Unadjusted OR (95% CI)** ^**b**^	***P-*value**	**Adjusted OR (95% CI)** ^**b**^	***P-*value**
Loses sleep over worry	36	33	57	52	0.5 (0.3, 0.8)	.004	0.5 (0.3, 0.9)	.021
Nervous, anxious, or on edge	36	33	58	53	0.4 (0.3, 0.8)	.003	0.4 (0.2, 0.8)	.010
Unable to cope with life	17	16	35	32	0.4 (0.2, 0.8)	.005	0.4 (0.2, 0.8)	.007
Easily discouraged by failure	58	53	51	46	1.3 (0.8, 2.2)	.346	1.7 (0.9, 3.2)	.070
Not positive about the future	8	7	17	16	0.4 (0.2, 1.0)	.061	0.5 (0.2, 1.3)	.127
Feels lonely	22	20	28	26	0.7 (0.4, 1.4)	.335	0.8 (0.4, 1.6)	.477
No-one to turn to at home for support	6	6	17	16	0.3 (0.1, 0.8)	.020	0.4 (0.1, 1.2)	.112
No-one to talk to outside family	13	12	18	16	0.7 (0.3, 1.5)	.334	0.8 (0.4, 1.9)	.678
If she help others, they will not help her back	9	8	17	16	0.5 (0.2, 1.1)	.100	0.6 (0.2, 1.5)	.279
Not participating in community or women’s group	63	57	70	64	0.8 (0.4, 1.3)	.335	0.8 (0.4, 1.4)	.402
Not in contact with friends or family	21	19	35	32	0.5 (0.3, 0.9)	.032	0.5 (0.2, 0.9)	.029
		
	**DIL**		**MIL**		**Odds of outcome for DIL relative to MIL, by logistic regression**			
Would cope with life better if she had:	***F***	**%**	***F***	**%**	**Unadjusted OR (95% CI)** ^**b**^	***P-*value**	**Adjusted OR (95% CI)** ^**b**^	***P-*value**
More education^**c**^	105	96	2	2	1134 (215, 5974)	<.001	4400 (285, 67 729)	<.001
Married at older age	90	82	100	91	0.5 (0.2, 1.0)	.054	0.4 (0.2, 0.9)	.038
Had first child at an older age	73	66	83	76	0.6 (0.4, 1.2)	.139	0.6 (0.3, 1.2)	.165

*n* = 110. Overall stress score derived by adding the 11 lived experience questions listed below. DIL, daughter-in-law; MIL, mother-in-law; PCA, principal component analysis; SD, standard deviation; △, difference; CI, confidence interval; F, frequency; %, percentage; OR, odds ratio.

a Linear regression. Adjusted models adjust for dyad’s age and marriage age, individual woman’s education and household assets.

b Logistic regression. Adjusted models adjust for dyad’s age and marriage age, individual woman’s education and household assets.

c See discussion for explanation of large OR.

A similar proportion among both groups felt lonely, though few had no one to speak to outside their family. Just over half of both groups were not participating in community or women’s groups.

Although 94% of MIL had no formal education, a negligible proportion (2%) felt that more education would help them cope with life better, in contrast to the vast majority of DIL (96%). Compared to MIL, DIL were less likely to feel that later marriage would have improved their coping ability, but the majority of both groups felt having their first child at a later age would have improved their ability to cope.

**Figure 2:**
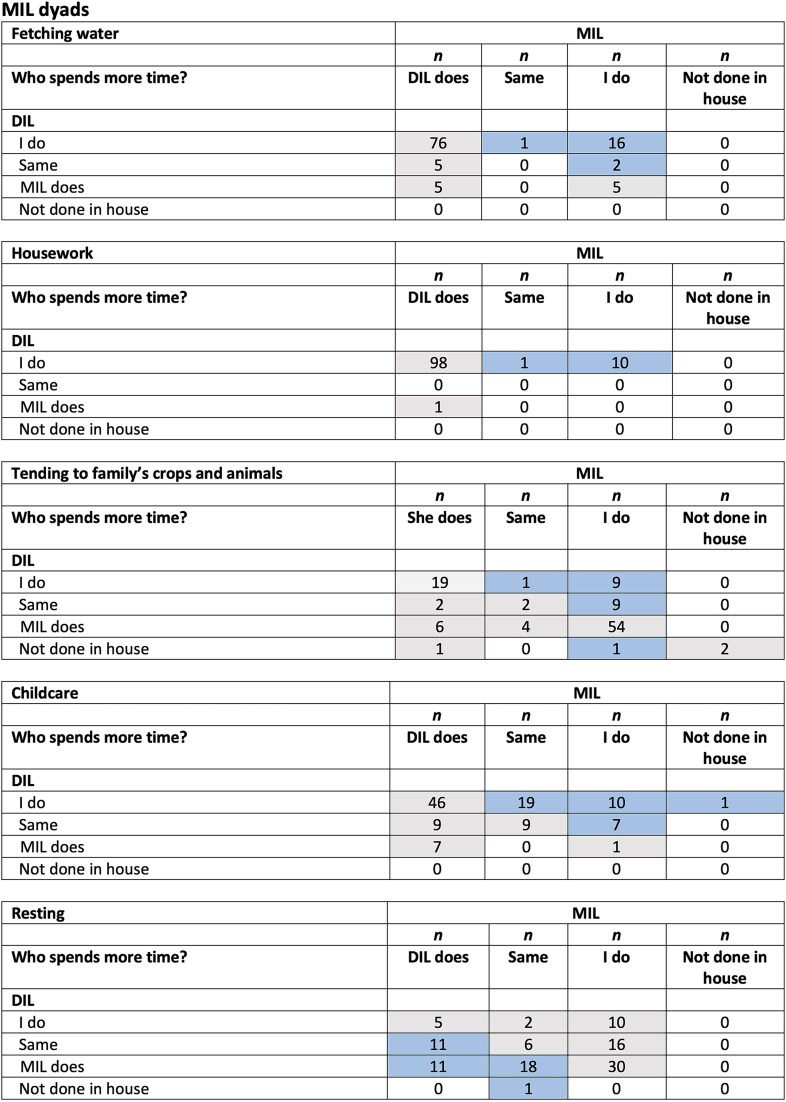
Perceived mutual effort in tasks performed by co-resident DIL and MIL dyads. Lighter shaded cells indicate inferred agreement or generosity between DIL and MIL over who spends more time on each of the five tasks: fetching water, housework, tending to the family’s crops and animals, childcare, and resting. Darker shaded cells indicate inferred disagreement or conflict over who does the task. *n*, number. *n* = 110. DIL, daughter-in-law; MIL, mother-in-law.

Figure 2 examines inferred conflict between DIL and MIL by visualizing their level of agreement over attributing the time spent by each on five unpaid care tasks. Seventy-six dyads (69%) agreed on who fetched water, overwhelmingly the DIL. Ninety-eight dyads (89%) agreed on who spent more time doing housework (always the DIL). Seventy-five dyads (68%) agreed on who did farming, of which nearly 75% said it was the MIL. Fifty-six dyads (51%) agreed on who did the childcare, of which 82% said it was the DIL. Only 41 dyads (37%) agreed on who spent more time resting—mostly the MIL. In some cases, when the answer did not indicate agreement, both women reported that the other did a greater share of the activity (or, in the case of rest, a lower share), indicating generosity over who performed the activity.

Frequencies of conflict (when one of the women reported doing more than the other recognized), and harmony (when they either agreed who did the most, or acknowledged a greater contribution by their counterpart), across the five tasks are given in Table S1. The most common conflict was over who rested more (37%) and did more childcare (34%). There were fewer disagreements over who fetched water (17%), did the housework (10%) and farming (18%). Overall, 66% of households showed conflicting perceptions between DIL and MIL over one or more tasks, while the remaining 34% were categorized as having no conflict.

The following analyses focus on childcare and resting, the most common forms of conflict between DIL and MIL. Only a minority of households (13%) reported experiencing both these disagreements (Table S2), while 43% reported experiencing neither. Households reporting these forms of conflict did not differ in terms of food security; however, conflict over resting was more common in households with greater wealth (Table S3). Conflict over childcare was more common among households with more educated DILs and those who had been married at an older age, while conflict over resting was more common among households where both MIL and DIL were younger and where there was a shorter age gap between the two women (Table S4).

**Figure 3:**
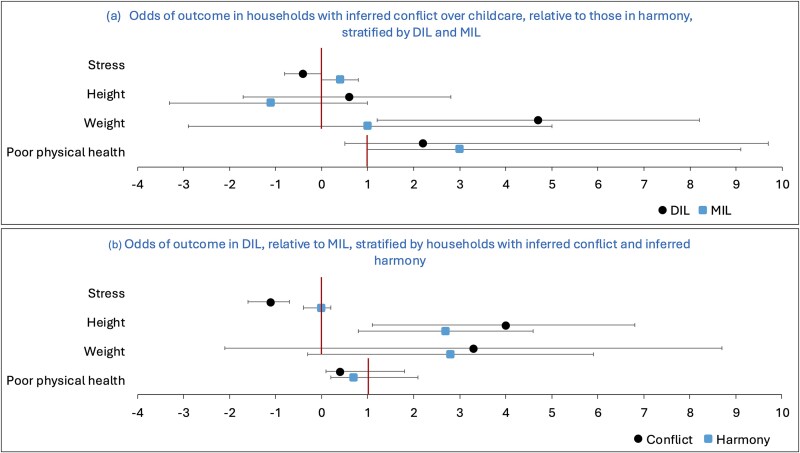
Health and anthropometric outcomes in households, illustrating (a) differences between households with inferred conflict over childcare relative to harmony, presented separately for DIL and MIL, and (b) differences of DIL relative to MIL, presented separately for households with inferred stress or harmony. Values are from multivariable linear regression models of stress, height, and weight (β, 95% CI, with reference group shown by a vertical line at 0) and multivariable logistic regression models of poor physical health (aOR, 95% CI, with reference group shown by a vertical line at 1) in households with inferred conflict over childcare (Tables S7 and S8). All models adjust for dyad’s age and marriage age, individual women’s education and household assets. In (a), circles indicate DIL and squares MIL. MIL is the reference group, indicated by the vertical lines. In (b), circles indicates conflict households and squares harmony households. Harmony households are the reference group, indicated by the red lines. DIL, daughter-in-law. MIL, mother-in-law.

Figure 3 shows results from adjusted regression models of stress, height, weight, and poor physical health by (a) differences in outcomes between households with inferred conflict vs harmony over childcare, stratified by DIL and MIL and (b) differences between DIL and MIL in households with inferred conflict vs harmony over childcare. Numerical values are given in Tables S7 and S8. Table S5 describes the health and nutritional status of co-resident DIL and MIL dyads in households with inferred conflict vs harmony over childcare and Table S6 for inferred conflict over resting.

Compared to DIL living in harmony households, DIL in households with inferred conflict over childcare reported being less stressed, and were heavier, but not taller, though they were more likely to report poor physical health. Conversely, compared to MIL living in harmony households, MIL in conflict households reported being more stressed, were more likely to have poor physical health, and were shorter.

Neither the total number of DIL children, the number of sons and daughters, nor the sex ratio of the child sample, was predictors of conflict over childcare (Table S9). All odds ratios were close to 1 with relatively wide CIs, *P* > .4 for all associations. For resting, there was weak evidence that the odds of conflict was lower in households with more children (OR 0.7, 95% CI 0.4, 1.3) or with more daughters (OR 0.6, 95% CI 0.2, 1.3); however, the child sex ratio showed negligible association (OR 0.9, 95% CI 0.4, 1.9) (Table S10).

In households with conflict over childcare, DIL reported being less stressed than MIL, and were taller and heavier though the weight difference had a wide CI. They were also less likely to report poor physical health. Conversely, in harmony households, there were far smaller differences in stress between the DIL and MIL, but DILs were again taller and heavier than the MIL, and had lower odds of reporting poor physical health.

Figure S1 shows broadly similar patterns were apparent in households reporting conflict over resting, except for height and weight, which were highest for DIL in harmonious households (Tables S11 and S12).

## DISCUSSION AND IMPLICATIONS

We aimed to shed new light on the MIL–DIL relationship in lowland Nepal, in a society where it forms a key part of women’s social niche. We aimed to evaluate the balance of power, using markers of physical and mental health as outcomes, and to test whether conflict was more or less likely in households with more children or daughters. Based on previous work, we expected to find that senior MIL had more agency and control over household dynamics and hence better nutritional status and mental health than junior DIL. However, our results did not support most of our hypotheses.

Maithili society is characterized by a household eating hierarchy, whereby younger adult women (DIL) have primary responsibility over preparing food, but typically eat last [31]. This is a common custom in South Asia. We found that DIL had better self-reported appetite, lower frequency of weight loss and better nutritional status, despite being below MIL in the eating order. Our results differ from previous research in this setting, which suggested that pregnant DIL (on average younger than our median sample age) were at greater risk of dietary micronutrient inadequacy and thinness compared to their MIL [31]. The DIL in our sample are no longer recently married, and their average age was > 30 years, suggesting they may have consolidated their position in the household. It is also possible that despite usually eating before DIL, MIL may feel obliged to leave a good quantity of food for DIL, who may still be reproducing, such that MIL voluntarily restrict their own intake. The MIL’s appetite might also reduce in alignment with falling caloric requirements with age and/or their lower mood.

Also contrary to our hypothesis, we found that MIL reported worse mental health than DIL. Underlying factors included greater loss of sleep over worry, and lower social support. In high-income countries, mental health shows a U-shaped association with age with worst outcomes in middle-age [35], while Gurven et al. reported that subjective life satisfaction rose with age in Nepal [26]. Our findings are inconsistent with both of these scenarios, and indicate that the experience of ageing in this setting needs further research. Although widows might be at particular risk of reduced social support and poor mental health [36], the 22 widows in our sample did not confirm this hypothesis. Another possibility is that differences in human capital might contribute. In this context, MIL attributed little benefit to education, whereas almost all DIL felt that more education would have helped them cope with life. Most likely, this generational contrast (reflecting the very large odds ratios in Table 3) was due to DIL being able to value an experience that either they had (*n* = 36), or their peers had had, whereas MIL (of whom only 6 had any education) generally lacked understanding of how education could have helped them. Both groups of women felt they would have benefitted from later marriage, though this was more common among MILs, likely reflecting their earlier age at marriage compared to DIL. Both groups of women also felt that they would have benefitted from having their first child at a later age.

Unpaid care work is not just under-recognized in academia and policy but also a source of tension among women [37]. Our assessment of inferred conflict over household effort may be an underestimate, as even when both groups agreed on respective effort, the group working harder might resent it. Notably, the main sources of inferred conflict were not over activities demanding higher energy expenditure (farm-work, fetching water, and housework) but rather home-based activities requiring persistent attention (childcare) or indicating self-interest (resting). It is possible that the MIL assumed her DIL would take responsibility for childcare, having already reared her own children, and felt that her own input was not recognized. Households reporting these two forms of conflict did not have greater food insecurity, instead conflict over resting was more common in households with greater wealth (Table S3). Conflict over childcare was more common among households with more educated DIL and those who had been married at an older age, while conflict over resting was more common among households where both MIL and DIL were younger. This suggests that greater DIL agency may have made conflict over childcare more likely, while women in younger dyads may have been more willing to challenge each other over resting.

An alternative possibility is that household tension over childcare may vary in association with degree of genetic relatedness between MIL and the grandchildren. Paternal grandmothers contribute X-chromosome genes to their granddaughters but not to their grandsons, increasing their inclusive fitness with granddaughters [15]. Therefore, MILs with more granddaughters might have greater vested interests in contributing to childcare. However, we found no evidence that either the number of DIL children nor their sex ratio was associated with the odds of conflict over childcare. There was weak evidence that conflict over resting was less common in households with more children and more daughters; however, CIs of these models were wide and the findings for sex ratio conflicted with those for the number of daughters. However, our sample size was small relative to earlier work by Fox et al. [15], hence this issue merits further investigation in larger samples. We note that in this society, boys will likely remain in the household and produce their own offspring while caring for their parents, whereas girls will be married into other households. As grandsons become adults, they will provide care for their father (the MIL’s son) as he ages and becomes a grandfather. Males also inherently benefit from inheriting the household property, and can use this to promote investment in their children, who share alleles with the MIL. Daughters not only miss out on this opportunity, but cost the household the dowry required for their marriage. Sons will also care for their mother (the current DIL) as she ages and potentially becomes a MIL if the son marries. For all of these reasons, sons tend to be more highly valued by both generations of women in this society, and may receive more investment from both DIL and MIL. However, paternity uncertainty would undermine the MIL’s pay-offs, and we have speculated previously that one factor contributing to the frequency of early marriage in this population is the greater paternity certainty resulting [38] with fitness benefits to the son/husband and MIL.

The health outcomes of DIL relative to MIL were better in conflict households compared to harmony households. Moreover, for some outcomes (physical health, stress, and weight), DIL had significantly better outcomes if they lived in conflict compared to harmony households, whereas the pattern for MIL was the opposite, showing the worst outcomes in conflict households. For these outcomes, we therefore suggest that household dynamics may lead to health contrasts.

However, a different pattern may apply to anthropometric traits. Notably, there was greater disparity between MIL and DIL in both weight and height in conflict compared to harmony households. Since growth in height ceases during adolescence, this suggests that taller DIL may take advantage of their more dominant physical status to risk challenging the MIL. In a matrilineal society in India, Leonetti et al. also found that women’s height varied according to the composition of the household and the relative power status of the women [21]. However, the scenario for weight is unclear, as while heavier DIL might be more assertive, more assertive women might also gain greater access to food and become heavier.

Overall, our results suggest that despite the more senior position of MIL in the household hierarchy, DIL may hold the upper hand, as shown by their better mental health outcomes, especially in households where there is conflict. Being younger, DIL may inherently have better physical health, as was observed in all households, and this may promote their agency, as may their larger body size.

Our study had several strengths, including a wide range of outcomes spanning physical health, nutritional status, lived experience, and subsistence activities for both co-resident women. Among the limitations, our ratings of physical health and appetite were obtained by self-report, and hence may not relate to actual health. Our data on household effort were also obtained by self-report, rather than objective measures—for example, from accelerometry or time diaries, hence we do not know which of the two women actually worked harder. Similarly, our assessment of conflict was based on inference rather than direct report, though this also avoided the possibility of introducing tension when asking about such scenarios. However, we were interested in perceived differences in effort, hence self-assessed ratings were more appropriate than objective measurements for our study question. We lacked information on other factors shaping lived experience, agency, and conflict for both women, in particular information about men either living in, or migrated away from, the household. As only four DILs were pregnant, we did not adjust for this in anthropometric variables, and excluding them did not change the results. With cross-sectional data, we cannot determine whether lived experience determined conflict or vice versa, and can only suggest the most likely scenario.

## CONCLUSION

The relationship between DIL and MIL has often been reported to be characterized by conflict, though from a theoretical perspective, both women could cooperate to gain Darwinian fitness benefits. We found that DIL reported better physical and mental health, despite the senior position held by MIL in the household hierarchy. Divergent perceptions over some unpaid care work were associated with higher stress in MIL, but not DIL. The number of children in the household was not predictive of the likelihood of such conflict. Further research is required to understand the factors shaping the lived experience of co-resident women.

## Supplementary Material

Supplementary_tables_figures_eoag006

## Data Availability

The data used in this study are available upon reasonable request and through a data sharing agreement. Contact the senior author, Professor Jonathan C. K. Wells, jonathan.wells@ucl.ac.uk.
