# Supplementary material for: Daughters-in-law and mothers-in-law in lowland rural Nepal: The balance of power and health outcomes
Source: Evol Med Public Health. 2026 Mar 27;14(1):1–14. doi: 10.1093/emph/eoag006 (PMC13221951; doi:10.1093/emph/eoag006)
Supplement: Supplementary_tables_figures_eoag006 [file supplementary_tables_figures_eoag006.docx]

**Supplementary materials**

**Supplementary text S1. Description of household wealth index and food insecurity**

Individual context-specific variables used to assess wealth [1] through Principal component analysis [2] included: house wall and roof material, number of bedrooms in the house, toilet type, access to electricity, water source, and possession of motorbike and colour television. This approach, of deriving a wealth index through PCA is widely used in Nepal and other countries [2].

Food security was assessed by the widely used Household Food Insecurity Scale [3]. Nine questions were asked to DIL:

1. In the past four weeks, did you worry that your household would not have enough food?
2. In the past four weeks, were you or any household member not able to eat the kinds of foods you preferred because of a lack of resources?
3. In the past four weeks, did you or any household member have to eat a limited variety of foods due to a lack of resources?
4. In the past four weeks, did you or any household member have to eat some foods that you really did not want to eat because of a lack of resources to obtain other types of food?
5. In the past four weeks, did you or any household member have to eat a smaller meal than you felt you needed because there was not enough food?
6. In the past four weeks, did you or any household member have to eat fewer meals in a day because there was not enough food?
7. In the past four weeks, was there ever no food to eat of any kind in your household because of lack of resources to get food?
8. In the past four weeks, did you or any household member go to sleep at night hungry because there was not enough food?
9. In the past four weeks, did you or any household member go a whole day and night without eating anything because there was not enough food?

**References**

1. Saville NM, Manandhar DS, Wells JC. Trial Experience and Data Capture in the Low Birth Weight South Asia Trial, a Large Cluster-Randomised Controlled Trial in Lowland Nepal. London: UK: UCL Discovery, 2020.

2. Rutstein SO, Johnson K. The DHS Wealth Index. Calverton: USA: ORC Macro, 2004.

3. Coates J, Swindale A, Bilinsky P. Household Food Insecurity Access Scale (HFIAS) for Measurement of Food Access: Indicator Guide. Washington DC: USA: Food and Nutrition Technical Assistance Project, Academy for Educational Development, 2007.

**Supplementary text S2. Questions asked to DIL and MIL on their lived experience**

Responses to each of the 11 questions were binary: yes or no

In last 4 weeks,

1. have you lost sleep over worry?
2. have you been bothered by feeling nervous, anxious, or on the edge?
3. have you felt you can cope with whatever comes along?
4. have you been easily discouraged by failure?
5. have you felt positive about the future?
6. have you felt lonely?
7. have you felt that there is a person at home who you can ask to support you?
8. have you felt that there are people you can talk to outside of your family if you have a problem?
9. have you felt that if you help someone, this person will help you when you need it?
10. have you participated in a community or women's group?
11. have you been in contact with friends or family outside of your marital home?

Overall: We would like to ask you some questions about what is going on in your life right now…

1. How would you rate the amount of stress in your life?

- Multiple choice response, read out to participants, where only 1 response was possible:
  - not at all stressed
  - a little bit stressed
  - quite a bit stressed or
  - extremely stressed

1. Do you think you could manage life better if you had…

- Multiple choice response, read out to participants, where >1 response was possible:
  - more education
  - married at an older age
  - had your first child at an older age
  - other (women were asked to specify)

**Supplementary Table S1. Inferred conflict over tasks in co-resident DIL and MIL households**

|  | **Conflict** | | **Harmony** | |
| --- | --- | --- | --- | --- |
| **Task** | **F** | **%** | **F** | **%** |
| Fetching water | 19 | 17 | 91 | 83 |
| Housework | 11 | 10 | 99 | 90 |
| Farming | 20 | 18 | 90 | 82 |
| Childcare | 37 | 34 | 73 | 66 |
| Resting | 41 | 37 | 69 | 63 |

*n*=110. DIL, daughter-in-law. MIL, mother-in-law. F, frequency. %, percentage.

**Supplementary Table S2. Consistency in inferred conflict over childcare and resting in co-resident DIL and MIL households**

|  | **Resting** | | | |
| --- | --- | --- | --- | --- |
|  | **Conflict** | | **Harmony** | |
| **Childcare** | **F** | **%** | **F** | **%** |
| Conflict | 15 | 13 | 22 | 20 |
| Harmony | 26 | 24 | 47 | 43 |

Numbers sum to 110 dyads. DIL, daughter-in-law. MIL, mother-in-law. F, frequency. %, percentage.

**Supplementary Table S3. Characteristics of households in inferred conflict over childcare and resting**

|  | **Childcare** | | | | | **Resting** | | | | |
| --- | --- | --- | --- | --- | --- | --- | --- | --- | --- | --- |
|  | **Conflict** | | **Harmony** | |  | **Conflict** | | **Harmony** | |  |
|  | **F** | **%** | **F** | **%** | ***p*-value^1^** | **F** | **%** | **F** | **%** | ***p*-value^1^** |
| Assets |  |  |  |  | 0.449 |  |  |  |  | 0.046 |
| 1: Poorest | 8 | 22 | 13 | 18 |  | 6 | 15 | 15 | 22 |  |
| 2 | 6 | 16 | 22 | 30 |  | 8 | 19 | 20 | 29 |  |
| 3 | 12 | 32 | 18 | 25 |  | 9 | 22 | 21 | 30 |  |
| 4: Richest | 11 | 30 | 20 | 27 |  | 18 | 44 | 13 | 19 |  |
|  |  |  |  |  |  |  |  |  |  |  |
| Household food security |  |  |  |  | 0.368 |  |  |  |  | 0.626 |
| Yes | 26 | 70 | 57 | 78 |  | 32 | 78 | 51 | 74 |  |
| No | 11 | 30 | 16 | 22 |  | 9 | 22 | 18 | 26 |  |

*n*=110. F, frequency. %, percentage. ^1^Chi-squared test.

**Supplementary Table S4. Characteristics of co-resident DIL and MIL dyads in households in inferred conflict over childcare and resting**

|  | **Childcare** | | | | | | **Resting** | | | | | |
| --- | --- | --- | --- | --- | --- | --- | --- | --- | --- | --- | --- | --- |
|  | **Conflict** | | **Harmony** | | **Difference,**  **Conflict vs Harmony** | | **Conflict** | | **Harmony** | | **Difference,**  **Conflict vs Harmony** | |
|  | **Mean** | **SD** | **Mean** | **SD** | **△ (95% CI)** | ***p*-value^1^** | **Mean** | **SD** | **Mean** | **SD** | **△ (95% CI)** | ***p*-value^1^** |
| Age (y) |  |  |  |  |  |  |  |  |  |  |  |  |
| DIL | 30.3 | 3.3 | 31.6 | 4.6 | -1.3 (-2.9, 0.4) | 0.141 | 30.0 | 3.2 | 31.8 | 4.6 | -1.8 (-3.3, -0.4) | 0.015 |
| MIL | 58.9 | 7.4 | 60.8 | 7.8 | -1.9 (-5.0, 1.1) | 0.210 | 57.6 | 6.5 | 61.7 | 7.9 | -4.1 (-6.8, 1.3) | 0.004 |
| Age difference (y) | 28.5 | 6.9 | 29.2 | 6.4 | -0.7 (-3.3, 1.9) | 0.605 | 27.6 | 6.2 | 29.8 | 6.7 | -2.2 (-4.8, 0.3) | 0.088 |
| Marriage age (y) |  |  |  |  |  |  |  |  |  |  |  |  |
| DIL | 15.7 | 1.8 | 15.0 | 1.6 | 0.7 (0.1, 1.4) | 0.038 | 15.4 | 1.5 | 15.1 | 1.8 | 0.3 (-0.4, 0.9) | 0.394 |
| MIL | 12.1 | 2.8 | 12.5 | 2.4 | -0.4 (-1.4, 0.7) | 0.474 | 12.6 | 2.3 | 12.2 | 2.7 | 0.4 (-0.6, 1.4) | 0.428 |
| Education (y) |  |  |  |  |  |  |  |  |  |  |  |  |
| DIL | 3.8 | 4.2 | 1.8 | 3.3 | 2.0 (0.4, 3.6) | 0.015 | 2.9 | 4.1 | 2.2 | 3.5 | 0.6 (-0.8, 2.1) | 0.400 |
| MIL | 0.4 | 1.3 | 0.1 | 0.6 | 0.3 (-0.1, 0.8) | 0.161 | 0.1 | 0.3 | 0.3 | 1.1 | -0.3 (-0.6, 0.1) | 0.065 |

Childcare: conflict *n*=37, harmony *n*=73. Resting: conflict *n*=41, harmony *n*=69. DIL, daughter-in-law. MIL, mother-in-law. y, years. SD, standard deviation. △, Difference. CI, Confidence Interval. ^1^Independent samples t-test.

**Supplementary Table S5. Description of health and nutritional status of co-resident DIL and MIL dyads in households with inferred conflict vs harmony over childcare**

|  | **Conflict** | **Harmony** | **△ (95% CI)^1^**  **Conflict vs harmony** |
| --- | --- | --- | --- |
| **Stress**  (PCA score: lost sleep, anxious) | **Mean (SE)** | **Mean (SE)** |  |
| DIL | -0.5 (± 0.1) | -0.0 (± 0.1) | -0.5 (-0.8, -0.1) |
| MIL | 0.5 (± 0.2) | 0.1 (± 0.1) | 0.4 (-0.0, 0.5) |
| **△ (95% CI)^1^, DIL relative to MIL** | -0.9 (-1.3, -0.6) | -0.1 (-0.4, 0.2) |  |
|  |  | | |
| **Height** (cm) |  | | |
| DIL | 152.1 (± 0.8) | 151.2 (± 0.6) | 0.9 (-1.1, 3.0) |
| MIL | 147.8 (± 0.0) | 148.4 (± 0.7) | -0.7 (-2.7, 1.4) |
| **△ (95% CI)^1^, DIL relative to MIL** | 4.4 (2.1, 6.6) | 2.8 (0.98, 4.6) |  |
|  |  | | |
| **Weight** (kg) |  | | |
| DIL | 52.4 (± 1.5) | 47.8 (±0.8) | 4.6 (1.4, 7.8) |
| MIL | 46.9 (± 1.6) | 45.2 (± 1.2) | 1.7 (-2.3, 5.7) |
| **△ (95% CI)^1^, DIL relative to MIL** | 5.3 (0.8, 9.4) | 2.6 (-0.3, 5.4) |  |
|  |  | | |
| **Poor physical health**  (self-reported) | **Percentage** | **Percentage** | **OR (95% CI)^2^, Conflict vs harmony** |
| DIL | Poor: 13%; Good: 87% | Poor: 8%; Good: 92% | 1.8 (0.5, 6.2) |
| MIL | Poor: 27%; Good: 73% | Poor:14% Good: 86% | 2.3 (0.9, 6.3) |
| **OR (95% CI)^2^, DIL relative to MIL**^2^ | 0.4 (0.1, 1.4) | 0.6 (0.2, 1.6) |  |

PCA, Principal component analysis score of stress. cm, centimetre. Kg, kilogram. SE, standard error. △, Difference. CI, Confidence Interval. ^1^Independent samples t-test. ^2^Chi-squared test.

**Supplementary Tables S6. Linear regression of co-resident DIL and MIL dyads in households with inferred conflict vs harmony over childcare**

|  | **Stress** (PCA score: lost sleep, anxious) | | **Height** (cm) | | **Weight** (kg) | |
| --- | --- | --- | --- | --- | --- | --- |
|  | **Model 1**  **Unadjusted** | **Model 2**  **Adjusted**^1^ | **Model 1**  **Unadjusted** | **Model 2**  **Adjusted**^1^ | **Model 1**  **Unadjusted** | **Model 2**  **Adjusted**^1^ |
|  | **β (95% CI)** | **β (95% CI)** | **β (95% CI)** | **β (95% CI)** | **β (95% CI)** | **β (95% CI)** |
|  | **Difference in outcome between inferred conflict vs harmony households stratified by DIL and MIL**^2^ | | | | | |
| DIL | -0.5 (-0.8, -0.1) | -0.4 (-0.8, 0.0) | 0.9 (-1.1, 3.0) | 0.6 (-1.7, 2.8) | 4.6 (1.4, 7.8) | 4.7 (1.2, 8.2) |
| MIL | 0.4 (-0.02, 0.8) | 0.4 (0.0, 0.8) | -0.7 (-2.8, 1.5) | -1.1 (-3.3, 1.0) | 1.7 (-2.3, 5.7) | 1.0 (-2.9, 5.0) |
|  | **Difference between DIL and MIL in inferred conflict vs harmony households**^3^ | | | | | |
| Conflict | -0.9 (-1.3, -0.5) | -1.1 (-1.6, -0.7) | 4.4 (2.1, 6.6) | 4.0 (1.1, 6.8) | 5.5 (1.1, 10.0) | 3.3 (-2.1, 8.7) |
| Harmony | -0.1 (-0.4, 0.2) | 0.0 (-0.4, 0.3) | 2.8 (1.0, 4.5) | 2.7 (0.8, 4.6) | 2.6 (-0.3, 5.5) | 2.8 (-0.3, 5.9) |

PCA, principal component analysis score of stress. cm, centimetre. Kg, kilogram. CI, Confidence Interval. ^1^Adjusted for dyad’s age and marriage age, individual women’s education and household assets. ^2^Reference category is harmony household. ^3^Reference category is MIL.

**Supplementary Table S7. Logistic regression of poor physical health of co-resident DIL and MIL dyads in households with inferred conflict vs harmony over childcare**

|  | **Model 1**  **Unadjusted OR (95% CI)** | **Model 2**  **Adjusted OR (95% CI)**^1^ |
| --- | --- | --- |
|  | **Odds ratio of outcome in conflict relative to harmony households, stratified by DIL and MIL**^2^ | |
| DIL | 1.8 (0.5, 6.2) | 2.2 (0.5, 9.7) |
| MIL | 2.3 (0.9, 6.3) | 3.0 (1.0, 9.1) |
|  | **Odds ratio of outcome for DIL relative to MIL, stratified by conflict and harmony households**^3^ | |
| Conflict | 0.4 (0.1, 1.4) | 0.4 (0.1, 1.8) |
| Harmony | 0.6 (0.2, 1.6) | 0.7 (0.2, 2.1) |

OR, Odds ratio. CI, Confidence Interval. ^1^Adjusted for dyad’s age and marriage age, individual women’s education and household assets. ^2^Reference category is harmony household. ^3^Reference category is MIL.

**Supplementary Table S8. Description of health and nutritional status of co-resident DIL and MIL dyads in households with inferred conflict vs harmony over resting**

|  | **Conflict** | **Harmony** | **△ (95% CI)^1^**  **Conflict vs harmony** |
| --- | --- | --- | --- |
| **Stress**  (PCA score: lost sleep, anxious) | **Mean (SE)** | **Mean (SE)** |  |
| DIL | -0.3 (± 0.2) | -0.1 (± 0.1) | -0.2 (-0.6, 0.2) |
| MIL | 0.1 (± 0.2) | 0.2 (± 0.1) | -0.1 (-0.5, 0.3) |
| **△ (95% CI)^1^, DIL relative to MIL** | -0.5 (-0.9, -0.0) | -0.4 (-0.7, -0.0) |  |
|  |  | | |
| **Height** (cm) |  | | |
| DIL | 152.0 (± 0.9) | 151.2 (± 0.6) | 0.7 (-1.3, 2.8) |
| MIL | 149.1 (± 0.8) | 147.7 (± 0.7) | 1.4 (-0.7, 3.5) |
| **△ (95% CI)^1^, DIL relative to MIL** | 2.9 (0.5, 5.2) | 3.5 (1.8, 5.3) |  |
|  |  | | |
| **Weight** (kg) |  | | |
| DIL | 47.5 (± 1.2) | 50.5 (±1.0) | -3.0 (-6.1, 0.2) |
| MIL | 45.5 (± 1.6) | 45.9 (± 1.2) | -0.5 (-4.4, 3.4) |
| **△ (95% CI)^1^, DIL relative to MIL** | 2.1 (-1.8, 5.9) | 4.5 (1.4, 7.7) |  |
|  |  | | |
| **Poor physical health**  (self-reported) | **Percentage** | **Percentage** | **△ (95% CI)^1^**  **Conflict vs harmony** |
| DIL | Poor: 7%; Good: 93% | Poor: 12%; Good: 88% | 0.6 (0.2, 2.4) |
| MIL | Poor: 20%; Good: 80% | Poor:17% Good: 83% | 1.2 (0.4, 3.1) |
| **OR (95% CI)^2^, DIL relative to MIL**^2^ | 0.3 (0.1, 1.3) | 0.6 (0.2, 1.6) |  |

PCA, principal component analysis score of stress. cm, centimetre. Kg, kilogram. SE, standard error. △, Difference. CI, Confidence Interval. ^1^Independent samples t-test. ^2^Chi-squared test.

**Table S9**. **Logistic regression of children in conflict vs harmony over childcare households**

|  |  | Unadjusted OR  (95% CI) | Adjusted OR  (95% CI)^1^ |
| --- | --- | --- | --- |
| Model 1 | Total number of children | 0.8 (0.6, 1.3) | 1.2 (0.7, 2.2) |
| Model 2 | Total number of girls | 0.9 (0.5, 1.5) | 1.2 (0.6, 2.6) |
|  | Total number of boys | 0.8 (0.4, 1.8) | 1.1 (0.4, 3.3) |
| Model 3 | Sex ratio (girls/boys) | 0.9 (0.6, 1.7) | 1.0 (0.5, 2.0) |

OR, Odds ratio. CI, Confidence interval. ^1^Adjusted for dyad’s age and marriage age, individual women’s education and household assets.

**Table S10**. **Logistic regression of children in conflict vs harmony over resting households**

|  |  | Unadjusted OR  (95% CI) | Adjusted OR  (95% CI)^1^ |
| --- | --- | --- | --- |
| Model 1 | Total number of children | 0.7 (0.4, 1.0) | 0.7 (0.4, 1.3) |
| Model 2 | Total number of girls | 0.5 (0.3, 1.0) | 0.6 (0.3, 1.3) |
|  | Total number of boys | 0.5 (0.2, 1.1) | 0.6 (0.2, 1.7) |
| Model 3 | Sex ratio (girls/boys) | 0.9 (0.5, 1.5) | 0.9 (0.4, 1.9) |

OR, Odds ratio. CI, Confidence interval. ^1^Adjusted for dyad’s age and marriage age, individual women’s education and household assets.

**Supplementary Figure S1. Health and nutritional status in households with inferred conflict over resting**

**
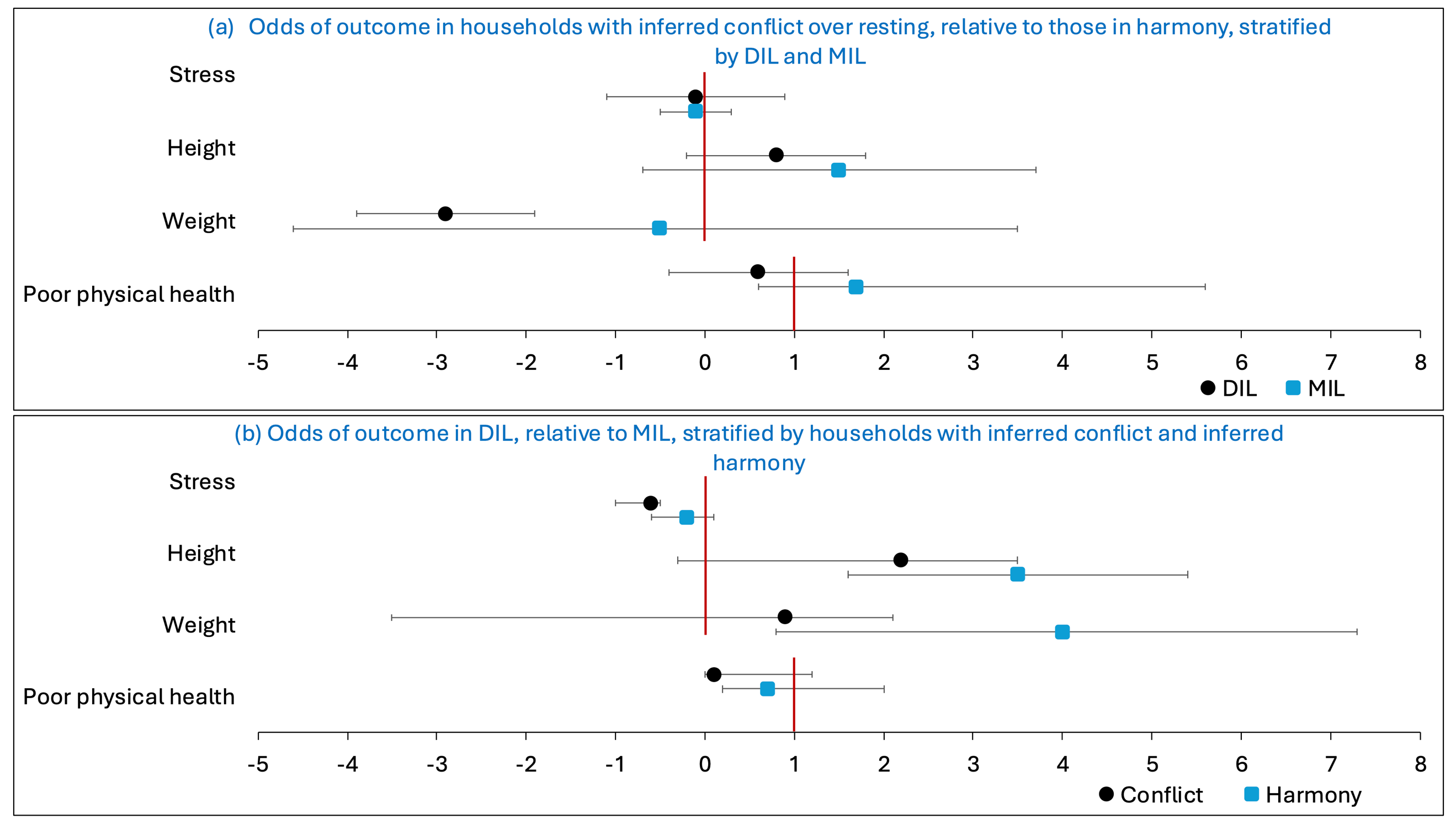
**

The Figure illustrates results from adjusted linear regression models of stress, height and weight (β, 95% CI, with reference group shown by red line at 0) and adjusted logistic regression models of poor physical health (aOR, 95% CI, with reference group shown by red line at 1) in households with inferred conflict over childcare (**Tables S9-S10**). Models adjust for dyad’s age and marriage age, individual women’s education and household assets. In Figure (a), black circles indicate DIL and blue squares MIL. MIL is the reference group, indicated by the red lines. In Figure (b), black circle indicates conflict households and blue squares harmony households. Harmony households are the reference group, indicated by the red lines. DIL, daughter-in-law. MIL, mother-in-law.

**Supplementary Table S11. Linear regression of co-resident DIL and MIL dyads in households with inferred conflict vs harmony over resting**

|  | **Stress** (PCA score: lost sleep, anxious) | | **Height** (cm) | | **Weight** (kg) | |
| --- | --- | --- | --- | --- | --- | --- |
|  | **Model 1**  **Unadjusted** | **Model 2**  **Adjusted**^1^ | **Model 1**  **Unadjusted** | **Model 2**  **Adjusted**^1^ | **Model 1**  **Unadjusted** | **Model 2**  **Adjusted**^1^ |
|  | β (95% CI) | β (95% CI) | β (95% CI) | β (95% CI) | β (95% CI) | β (95% CI) |
|  | **Difference in outcome between conflict vs harmony households stratified by DIL and MIL**^2^ | | | | | |
| DIL | -0.2 (-0.6, 0.2) | -0.1 (-0.5, 0.3) | 0.7 (-1.3, 2.7) | 0.8 (-1.3, 2.9) | -3.0 (-6.1, 002) | -2.9 (-6.3, 0.5) |
| MIL | -0.1 (-0.5, 0.3) | -0.1 (-0.5, 0.3) | 1.4 (-0.7, 3.5) | 1.5 (-0.7, 3.7) | -0.5 (-4.4, 3.4) | -0.5 (-4.6, 3.5) |
|  | **Difference between DIL and MIL in conflict vs harmony households**^3^ | | | | | |
| Conflict | -0.4 (-0.9, -0.0) | -0.6 (-1.0, -0.1) | 2.9 (0.5, 5.2) | 2.2 (-0.3, 4.8) | 2.1 (-1.8, 5.9) | 0.9 (-3.5, 5.2) |
| Harmony | -0.4 (-0.7, -0.1) | -0.2 (-0.6, 0.1) | 3.5 (1.8, 5.3) | 3.5 (1.6, 5.4) | 4.5 (1.4, 7.7) | 4.0 (0.8, 7.3) |

PCA, Principal component analysis score of stress. cm, centimetre. Kg, kilogram. CI, Confidence Interval. ^1^Adjusted for dyad’s age and marriage age, individual women’s education and household assets. ^2^Reference category is harmony household. ^3^Reference category is MIL.

**Supplementary Table S12. Logistic regression of poor physical health in conflict vs harmony over resting households**

|  | **Model 1**  **Unadjusted OR (95% CI)** | **Model 2**  **Adjusted OR (95% CI)**^1^ |
| --- | --- | --- |
|  | **Odds ratio of outcome in conflict relative to harmony households, stratified by DIL and MIL** ^2^ | |
| DIL | 0.6 (0.2, 2.4) | 0.6 (0.1, 2.8) |
| MIL | 1.2 (0.4, 3.1) | 1.7 (0.6, 5.6) |
|  | **Odds ratio of outcome for DIL relative to MIL, stratified by conflict and harmony households**^3^ | |
| Conflict | 0.3 (0.1, 1.3) | 0.1 (0.0, 1.2) |
| Harmony | 0.6 (0.2, 1.6) | 0.7 (0.2, 2.0) |

OR, Odds ratio. CI, Confidence Interval. ^1^Adjusted for dyad’s age and marriage age, individual women’s education and household assets. ^2^Reference category is harmony household. ^3^Reference category is MIL.
